# GLP-1 receptor agonists in IBD: exploring the crossroads of metabolism and inflammation

**DOI:** 10.3389/fimmu.2025.1610368

**Published:** 2025-07-15

**Authors:** Giulia Migliorisi, Roberto Gabbiadini, Arianna Dal Buono, Matteo Ferraris, Giuseppe Privitera, Lorenzo Petronio, Peter Bertoli, Cristina Bezzio, Alessandro Armuzzi

**Affiliations:** ^1^ IBD Center, Humanitas Research Hospital - IRCCS, Rozzano, Milan, Italy; ^2^ Department of Biomedical Sciences, Humanitas University, Milan, Italy

**Keywords:** IBD, GLP-1, GLP-1 receptor agonists, diabetes, obesity

## Abstract

Glucagon-like peptide-1 receptor agonists (GLP-1 RAs) represent a cornerstone in the treatment of diabetes and obesity and have emerged as a promising option for other metabolic disorders, including hepatic steatosis. Recent evidence highlights the direct and indirect anti-inflammatory properties of GLP-1, suggesting a potential additional therapeutic strategy for patients with inflammatory bowel disease (IBD). However, side effects of GLP-1 RAs, particularly those affecting the gastrointestinal system, may limit their use in patients with IBD. The rising prevalence of IBD worldwide and the ageing of the IBD population will likely increase the number of patients with metabolic comorbidities who may potentially benefit from a combination treatment with GLP-1 RAs. A profound comprehension of the physiological function of intestinal homeostasis and permeability is essential to more accurately evaluate the prospective application of GLP-1 RAs in patients with ongoing inflammation. While preclinical studies support this hypothesis, robust clinical evidence remains limited. This narrative review aims to provide a synthesis of current knowledge regarding the anti-inflammatory properties of GLP-1, with a particular focus on safety concerns and potential future directions for its use in IBD management.

## 1 Introduction

Inflammatory bowel disease (IBD), comprising Crohn’s disease (CD) and ulcerative colitis (UC), is a multifactorial, chronic, immune-mediated inflammatory disorder that affects the gastrointestinal system. The prevalence and incidence of IBD are rising worldwide, placing a significant burden on healthcare systems and social resources (1). Although significant progress in the development of immunotherapy over the past two decades (2), approximately 50% of IBD patients show response failure after initial advanced therapies, with response rates exhibiting a further decline for second- and third-line treatments (3, 4). As a result, the exploration of novel therapeutic strategies is crucial for improving IBD management.

A promising explanation could be represented by the complex interplay between inflammation and metabolism, which is still largely unknown in the field of IBD management. Recent evidence suggests that Westernized lifestyles, including dietary habits and sedentary behavior, could have contributed to the increasing global prevalence of IBD, particularly in newly industrialized countries (5, 6). Notably, high sugar intake has been identified as a potential risk factor for gut inflammation in preclinical studies, leading to colitis and a significant accumulation of inflammatory cells in mesenteric fat and lymph nodes in genetically susceptible mice (7).

GLP-1 receptor agonists (GLP-1 RAs) have gained attention due to their numerous effects on gut metabolism and immune regulation. By stimulating glucose-dependent insulin secretion and suppressing glucagon production, they are a cornerstone of the treatment of type 2 diabetes (DM2), particularly in patients with a higher risk of cardiovascular disease (8). Additionally, GLP-1 RAs have been approved for obesity treatment given their ability to delay gastric emptying, with enhanced satiety and reduction of energy intake (9). In addition, GLP-1 RAs have shown to present direct anti-inflammatory effects, by modulating immune cell signalling and preventing the release of reactive oxygen species (ROS) (10). Preclinical studies indicate that GLP-1 RAs may have an impact on gut microbiota composition (11) and contribute to the maintenance of intestinal mucosal barrier integrity, thereby reducing gut permeability (12). Furthermore, by addressing metabolic dysfunction in obese patients, whose prevalence is increasing in the IBD population (13), GLP-1 RAs could indirectly mitigate inflammation by decreasing the pro-inflammatory activity of adipose tissue, particularly visceral adipose one. The convergence of metabolic and inflammatory pathways suggests that GLP-1 RAs hold promise as an adjunctive therapy for IBD. Nevertheless, their therapeutic potentials are still subject of debate, particularly given their known gastrointestinal side effects (e.g., nausea, vomiting, and diarrhea), which may limit their application in IBD patients, especially those with clinically active disease (14).

This narrative review describes the complex interplay between GLP-1 signalling and intestinal inflammation, highlighting both the potential benefits and limitations of GLP1 RAs in IBD management, with a particular focus on their promising role in selected obese IBD patients.

## 2 GLP-1: a key player in metabolic homeostasis

GLP-1 is a 31 aminoacid-long peptide derived from proglucagon (15), which is produced in response to both nutritional and inflammatory stimuli. It is mainly produced by enteroendocrine L-cells, which are distributed throughout the gastrointestinal tract, with increasing density from the proximal jejunum to the colon (16). Additionally, GLP-1 is secreted by brainstem neuronal cells (17). The release of GLP-1 follows a biphasic pathway: an initial peak mediated by neural signalling, followed by a second phase which is triggered by direct mechanical stimulation of L-cells, as nutrients pass through the gut (18).

GLP-1 is best known for its role in glucose homeostasis. It enhances insulin secretion from pancreatic β-cells in a glucose-dependent manner, whilst simultaneously suppressing glucagon release from α-cells (19). It also promotes β-cell survival and proliferation, strengthening its role in metabolic regulation (20). Apart from its metabolic effects, GLP-1 plays a key role in gastrointestinal motility. It is the main mediator of the phenomenon known as the “ileal brake” (21), which delays both gastric emptying and small bowel transit, with resulting slowed nutrient absorption in direct proportion to carbohydrate intake (22, 23). This effect is primarily mediated by GLP-1 receptors (GLP-1Rs) located on myenteric neurons of the digestive system, involving nitrergic and cyclic adenosine monophosphate (cAMP)-dependent mechanisms (22, 24).

Notably, GLP-1 appears to be a key messenger of the gut-brain axis. In response to significant and/or high-nutrient meals, GLP-1 reaches high blood concentrations, exerting its effects on GLP-1Rs located in the area postrema, the nucleus tractus solitarius, and the hypothalamus. As a result, it induces satiety and regulates food intake (25). This effect is significantly more pronounced in patients undergoing GLP-1 RAs compared to the endogenous one (26), thus justifying the use of these drugs in the treatment of obesity. In support of their role in weight management, GLP-1Rs are expressed in adipose tissue, where they regulate the proliferation of pre-adipocyte cells and lipid homeostasis (27).

Apart from its metabolic effects, GLP-1 exerts a wide range of physiological actions. Its receptors are expressed in multiple tissues, including the heart, kidneys, lungs, and smooth muscle. The protective effects of GLP-1 RAs against cardiovascular events have been demonstrated in several studies, thus supporting their application in patients with heart failure with preserved ejection fraction and DM2 (28). These benefits primarily derived from improved glucose control, weight reduction, enhanced cardiac output, and lower blood pressure. Additionally, GLP-1 plays a protective role for the endothelium. It reduces atherosclerotic plaque formation, inhibits the expression of vascular adhesion molecules, and prevents LDL-induced immune cell adhesion (29–31). Notably, GLP-1Rs have also been found in the atrial cavities and in the sinoatrial node, still their precise physiological function remains unclear (32). Additionally, the therapeutic potentials of GLP-1 could extend to renal and hepatic health. Recent studies suggest that GLP-1 RAs could improve renal function in patients with chronic kidney disease (CKD) and DM2 (33, 34). Despite the absence of GLP-1Rs on hepatocytes, GLP-1 is involved in hepatic lipid and glucose metabolism, by contributing to fibrosis reversal and liver cells protection in patients with nonalcoholic steatohepatitis (NASH) undergoing GLP-1 RAs (35).

Taken together, GLP-1 is a major metabolic and regulatory hormone, with many potential therapeutic implications beyond glycemic control, encompassing cardiovascular protection, enhanced renal function, and the potential benefits in hepatic disease.

## 3 GLP-1’s anti-inflammatory properties

Recent scientific research has increasingly highlighted the potential anti-inflammatory role of GLP-1. This premise is reinforced by the discovery that different immune cells, including B and T lymphocytes, as well as myeloid-lineage cells such as monocytes, eosinophils, and neutrophils, express GLP-1Rs (36). Notably, GLP-1Rs are also expressed by intestinal intraepithelial lymphocytes (IELs), suggesting a role in immune homeostasis of the digestive system (36, 37). Moreover, studies conducted on animal models have shown that enteroendocrine L-cells increase the secretion of GLP-1 in response to inflammatory cytokines (e.g., interleukin-6 (IL-6) and lipopolysaccharide (LPS) (38)) and ischaemic injury (39). Furthermore, Kahlen et al. found that critically ill ICU patients presented significantly higher plasma GLP-1 levels versus healthy controls, which were directly correlated to increased inflammatory biomarkers such as IL-6). These results underscore a potential connection between GLP-1 and inflammatory processes (40). Current evidence suggests that GLP-1 has a regulatory role in both innate and adaptive immunity while also supporting intestinal barrier integrity and gut microbiota health.

### 3.1 Innate and adaptive immune response

In rat models of systemic inflammation, a GLP-1 RA, exendin, significantly lowered pro-inflammatory cytokine levels, including IL-1β, IL-6, TNF-α, and interferon-gamma (IFN-γ) (41). These effects are primarily mediated by the inhibition of NF-κB and mitogen-activated protein kinase (MAPK) pathways, both of which are associated with stress, inflammation, and apoptosis responses (10, 42). Interestingly, in the context of several *in vitro* studies, GLP-1 RAs have demonstrated the capacity of promoting an anti-inflammatory state by influencing immune cell differentiation. For instance, exenatide, the first GLP-1-analogue developed, demonstrated to promote human monocyte differentiation into alternatively activated M2 macrophages, leading to an increase in anti-inflammatory cytokines such as IL-10 while significantly reducing pro-inflammatory cytokines, including IL-6, TNF-α and IL-1β (43). Shiraishi et al. demonstrated that GLP-1/GLP-1R signalling plays a crucial role in activating signal transducer and activator of transcription 3 (STAT3), which directly promotes human M2 macrophage polarization while inhibiting classically activated M1 macrophages, known for their pro-inflammatory and tissue-destructive properties (44). In animal models, GLP-1/GLP-1R signalling has demonstrated to be involved in key macrophage functions such as phagocytosis and migration, though further research is needed to confirm these effects in humans (45). Additionally, both eosinophils and neutrophils express GLP-1R on their surface. Notably, eosinophils of asthmatic patients exhibit lower GLP-1Rs’ expression compared to healthy controls (46). The interaction between GLP-1 and its receptor on eosinophils has been shown to reduce the production of pro-inflammatory cytokines, including IL-4, IL-8 and IL-13 (47). Although the limited research on the role of GLP-1 in neutrophils, preliminary findings have shown that it may mitigate their activation, potentially reducing myocardial ischemic injury rodent models (48). In conclusion, the GLP-1/GLP-1R signalling plays a significant role in the balance of innate immunity, particularly in the polarisation of macrophages.

Furthermore, GLP-1 has been hypothesised to act as a mediator between innate and adaptive immune responses. In mice single-cell RNA sequencing identified a subpopulation of GLP-1R-positive memory T-cells that was mainly composed of exhausted CD8+ T cells: functionally, stimulation of the GLP-1R on these cells was found to mediate apoptosis and anergic signals, thereby suppressing effector T-cell function and the inflammatory response (49, 50). In humans, the GLP-1 pathways act as a modulator of a specific subset of T-cells, known as invariant natural killer T (iNKT) cells, and this activity might be responsible for the improvements of some immune-mediated disorders (such as psoriasis and suppurative hidradenitis) observed in obese patients treated with GLP-1 RAs (51–54). In mice, GLP-1 RA treatment also showed to inhibit the differentiation of T helper (Th) cells into Th1 and Th17 subsets and reduce the release of related proinflammatory cytokines, including IFN-y, TNF-α, and IL-17. Instead, GLP-1 promotes the polarization of Th2 and regulatory T (Treg) cells, increasing anti-inflammatory cytokines such as IL-10 and IL-5 (55, 56).

### 3.2 Intestinal mucosal barrier

IELs are a heterogeneous population of T cells located among intestinal epithelial cells (IECs), where they contribute to maintaining mucosal barrier integrity (57). A particular subset of IELs (Tαβ and Tγδ) has been found to be enriched with GLP-1Rs (37, 58). Their proximity to enteroendocrine L-cells suggests a potential contribution of lymphoid tissue in GLP-1/GLP-1R signalling, as mediator of L-cell proliferation (59, 60). Genetically modified *Glp1r* ^−/−^ mice displayed greater levels of epithelial damage in comparison to wild-type (WT) mice following inflammatory stimulation. Conversely, WT mice exhibited higher expression of antimicrobial and anti-inflammatory genes (37). Similarly, Wong et al. described that IEL-expressing GLP-1Rs play a crucial role in controlling gut inflammation of mice, by reducing IFN-γ production in IELs and promoting IEC survival and intestinal barrier integrity (38). Additionally, GLP-1 showed to directly stimulate murine Brunner’s glands to produce and release mucin, thereby strengthening the mucosal barrier (61). Furthermore, GLP-1Rs on IELs have been found to regulate the metabolic effects of GLP-1 in animal models by entrapping it, thereby reducing its systemic availability and lowering its plasma concentrations (62). Moreover, GLP-1 could be involved in mechanisms of growth and expansion of IECs, as the loss of GLP-1Rs on IELs has been associated with shorter and smaller intestines in mice (59, 63, 64).

The anti-inflammatory role of GLP-1 in the gut is not limited to the maintenance of IELs and L-cells but it is also interconnected with gut microbiota. Thus, several microbiota-derived metabolites have been found to stimulate enteroendocrine L-cells producing GLP-1. Short-chain fatty acids (SCFAs) such as butyrate, a bacterial metabolite produced from dietary fibre, can directly trigger GLP-1 release by binding to the membrane receptor GPR43 (65). Additionally, dietary protein-derived metabolites, including tryptophan-indole, as well as LPS from Gram-negative bacteria, directly promote the production of GLP-1 (66, 67). Interestingly, bile acid metabolites have a dual effect on GLP-1 secretion: they can both stimulate and inhibit its production (66). Furthermore, the ileum of germ-free and antibiotic-treated mice showed lower Glp1r gene expression compared to controls, with higher incretin effect resistance (68).

Both preclinical and clinical studies have demonstrated that GLP-1’s modulate the gut microbiota composition by delaying gastric emptying and altering luminal glucose (12). Zhao et al. observed that the gut microbiota of diet-induced obese (DIO) mice treated with liraglutide for four weeks displayed a similar phylogenetic composition in comparison to the start of the treatment, but was characterized by a significant decrease in microbial phenotypes associated with obesity (e.g., *Firmicutes Lachnospiraceae and Clostridiales*), alongside an increase in *Proteobacteria* (e.g., *Burkholderiales bacterium YL45*) and *Akkermansia muciniphila*—a species largely associated with high-fibre diets and mucosal barrier health (11, 69–71). Notably, the administration of GLP-1 RA resulted in an elevated *Firmicutes/Bacteroidetes* ratio and an increase in *Prevotella*, species that have been linked to lower inflammation (12, 70). Similar results were reported by Wang et al., where GLP-1 RA treatment had a notable impact on the abundance of weight-related microbial phylotypes, though no significant change in the *Firmicutes/Bacteroidetes* ratio was observed (72).

The findings concerning human gut microbiota remain controversial. Although liraglutide treatment led to a decrease in pro-inflammatory microbial species (e.g., *Escherichia–Shigella, Megamonas*, and *Bacillus*) in DM2 patients, no statistically significant differences were observed when compared to metformin treatment. However, a significant increase in *Bifidobacterium, Dialister*, and *Alistipes* was reported (73). A recent study conducted on 41 diabetic patients demonstrated that exposure to the GLP-1 RA dulaglutide over a 48-week period was associated with a substantial decrease in non-butyrate-producing *Firmicutes* (e.g., *Ruminococcus* and *Blautia*), accompanied by an increase in *Bacteroides, Lactobacillus*, and *Prevotella* (74).

**Figure 1:**
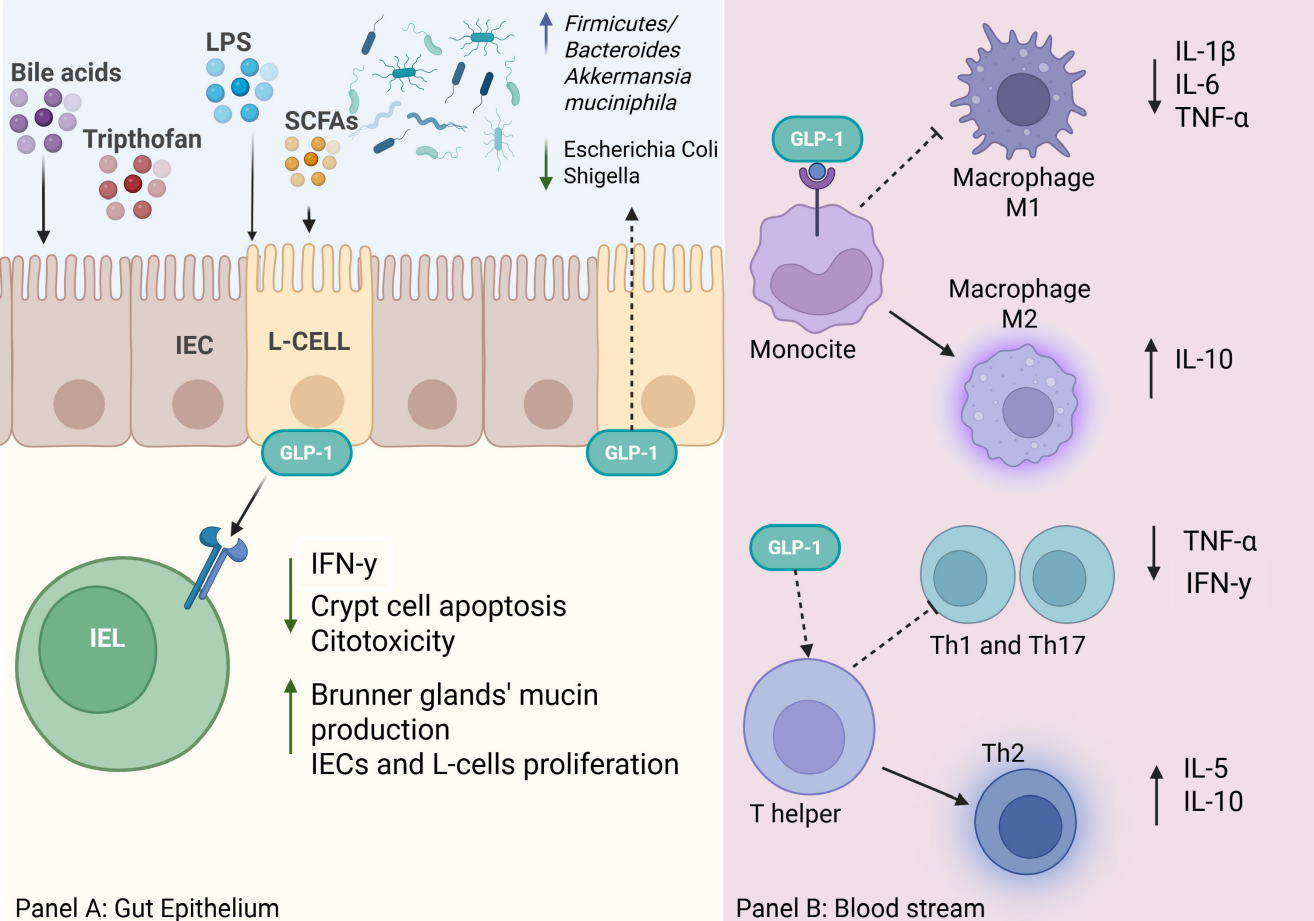
The image in panel **(A)** illustrates how various metabolites derived from diet and gut microbiota (bile acids, tryptophan, LPS, SCFAs) stimulate enteroendocrine L-cells in the intestinal mucosa to secrete GLP-1. GLP-1 acts both locally at the level of the intestinal epithelium and systemically through blood vessels and immune cells. GLP-1 reduces IFN-γ, crypt cell apoptosis, and cytotoxicity by intraepithelial lymphocytes (IELs) and at the same time, it stimulates the proliferation of intestinal epithelial cells (IECs) and L-cells, as well as mucin production by Brunner’s glands (37, 41, 49, 50, 61). Moreover, GLP-1 RAs are found to be associated with alteration of intestinal microbiota (11, 72). Panel B is a concise representation of the role of GLP-1 in immune responses. GLP-1 modulates monocytes/macrophages, promoting polarization toward M2 macrophages (anti-inflammatory), which release IL-10 and suppress pro-inflammatory cytokines (IL-1β, IL-6, TNF-α). Furthermore, it Influences indirectly T helper lymphocytes polarisation to Th2 cells and release of IL-5 and IL-10, contributing to a more tolerogenic immune response (41, 44). Overall, GLP-1 acts as an immuno-metabolic modulator, supporting intestinal barrier protection and a regulated inflammatory response.

In conclusion, GLP-1 contributes to gut homeostasis and inflammation control through its modulatory effects on immunity, epithelial cell proliferation, and microbial composition (**Figure 1**). Whilst emerging evidence suggests the extensive therapeutic potential of GLP-1, further studies are needed to fully elucidate its mechanisms and its clinical applications in gastrointestinal disorders.

## 4 Metabolic disorders and IBD

The prevalence of metabolic disorders, including DM2 and obesity, is increasingly rising in the IBD population. Approximately 15-40% of individuals diagnosed with IBD and an additional 20-40% are estimated to be obese and overweight respectively (13). IBD and metabolism are strongly interconnected (**Table 1**). Adipokine levels are frequently altered in IBD patients (75, 76). Leptin, a key regulator of satiety and appetite, is decreased in active IBD, probably due to exhaustion after transient overproduction related to TNF-α hyperactivity. In contrast, high levels of resistin levels have been found in active IBD, correlating with NF-κB pathway activation and increased secretion of TNF-α, IL-6, and IL-1β (77). Moreover, despite normal glycemic levels, elevated serum resistin has been linked to hyperinsulinemia in active IBD (78). IBD patients, present higher risk of developing insulin resistance, particularly those diagnosed with CD (79, 80). However, a recent study did not support this finding but rather attributed the increased risk of insulin resistance to the concomitant presence of metabolic dysfunction–associated steatotic liver disease (MASLD) (81). Furthermore, omentin-1, an anti-inflammatory adipokine that inhibits TNF-induced vascular inflammation, exhibit low levels in patients with active CD and UC (82), emphasising the profound connection between IBD and adipose tissue.

**Table 1:** IBD and metabolic disorders are closely interconnected.

Alterations of metabolism in IBD patients		Obesity impact on IBD patients	
Alteration of adipokines	• Decrease of leptin and omentin-1 • Increase of resistin	Onset of IBD	• Severe obesity and bariatric surgery are independent risk factors for IBD onset • Obesity in early adulthood increase risk of CD in elderly
Glycemic metabolism	• Hyperinsulinemia and insulin resistance in CD, although normal glycemia levels	IBD progression	• Higher risk of steroid, advanced therapies and surgery in UC • No risk of perianal and/or structuring complications in CD
Hepatic metabolism	• Increased risk of developing MASLD	Visceral fat	• Predictive factor of short-term postoperative recurrence in CD.

On one hand, IBD can lead to dysregulation of adipokines, shifting the balance towards a pro-inflammatory state, on the other hand obesity and higher BMI are associated with higher risk of developing IBD and worse clinical outcomes, particularly for UC. Although the association between IBD and insulin resistance and MASLD is controversial, visceral fat is gaining prominence as a predictive prognostic factor of postoperative recurrence in CD patients.

The impact of obesity on IBD onset and progression is an area of growing research, though evidence remains controversial. Severe obesity and bariatric surgery have been recognized as independent risk factors for the development of IBD (83). Additionally, obesity in early adults has been linked to a substantially increased risk of CD onset in the elderly (84). A recent propensity-matched cohort study also identified obesity as a risk factor for corticosteroid use, therapy escalation, and colectomy in UC patients (85). However, no increased risk of perianal or stricturing complications has been observed in CD patients (86, 87). Interestingly, a retrospective analysis of 202 UC patients showed that higher BMI was inversely related to disease severity and IBD extent (88). Nonetheless, BMI was directly associated with higher risk of severe hospitalization, longer hospital stays and increased surgical intervention rates, mainly due to metabolic comorbidities (89). The discrepancies may be attributed to the limitations of BMI as a lone evaluator of metabolic disorders. A recent cohort study involving 200 IBD patients identified visceral adiposity, rather than BMI, as a predictive risk factor for a shorter time to IBD flare, particularly for CD patients (90). Additionally, recent data recognized visceral fat as a risk factor for postoperative recurrence in CD patients (76).

### 4.1 The role of GLP-1 RAs in IBD

The previously mentioned intrinsic connection between metabolism and the inflammatory response led researchers to investigate the role of GLP-1 modulation in the management of IBD. Evidence suggests that IBD pathogenesis is closely linked to gut failure in controlling inflammation, with enteroendocrine cells (EECs) playing a pivotal role in the process (91). In this regard, TNF-α has been demonstrated to trigger the NF-κB pathway in EECs, which are a target of the GLP-1/GLP-1R pathway. This, in turn, results in the production of IL-17C, a process that contributes to the propagation of inflammation in individuals suffering from IBD (92). Preclinical models found that GLP-1 RAs reduce intestinal inflammation in dextran sulfate sodium (DSS)-induced colitis, by increasing IL-22 production by colonic IELs and several beneficial bacteria, including *Firmicutes, Proteobacteria and Lactobacillus reuteri* (93). Furthermore, in human samples, GLP-1R was deregulated in IBD active biopsies (94), with elevated GLP-1 plasmatic levels being associated with severe active disease (95). Anecdotal evidence suggests that GLP-1 RAs may be beneficial in IBD treatment. A case report documented clinical remission in a 42-year-old UC patient following liraglutide administration for obesity treatment (96). This lends further weight to prospective investigation of the benefits and safety of GLP-1 RAs in the real world which are resumed in **Table 2**.

**Table 2:** This table compiles current knowledge evaluating the impact of GLP-1 RAs on disease outcomes and safety in IBD patients.

Reference	Study design	Population	Key findings/outcome	Safety notes
Jeffrey et al. (96)	Case report	42-year-old with UC	Clinical remission after liraglutide treatment for obesity	Not specified
Desai et al. (97)	Retrospective cohort study	IBD with DM2	Significantly reduced risk of surgery	Not specified
Nielsen et al. (98)	Retrospective cohort study	IBD with DM2	Lower risk of corticosteroid use and hospitalization	Not specified
Gorelik et al. (99)	Retrospective cohort study	IBD with DM2 and/or obesity	Significantly reduced hospitalisation rates; benefit limited to obese subgroup	10% of GI adverse events
Sehgal et al. (100)	Retrospective cohort study	IBD obese patients	Significantly reduced CRP, near-significant reduction of fecal calprotectin	Not specified
Levine et al. (101)	Retrospective cohort study	IBD with DM2 or obesity	No significant difference in remission or escalation; CRP improved	No increased rate of IBD exacerbation
St-Pierre et al. (102)	Retrospective observational cross-sectional cohort study	Non-diabetic patients with IBD	No significant alterations in inflammatory markers	Not specified
Clarke et al. (103)	Retrospective cohort study	IBD obese patients	IBD did not reduce weight loss or affect anti-TNF-α synergy	Reduced risk of diarrhea/nausea, increased constipation rate
Anderson et al. (104)	Retrospective cohort study	IBD obese patients	Mild GI symptoms, no major disease activity change	Well tolerated
Ramos Belinchon et al. (105)	Retrospective case series	IBD obese patients	No major disease activity change	Well tolerated
Desai et al. (106)	Retrospective cohort study	IBD obese patients	Semaglutide most effective for weight loss;	Similar GI adverse events risk than general population

Overall, GLP-1 RAs show potential benefits including reduced hospitalization, lower inflammatory markers, and effective weight loss, particularly in obese patients. While gastrointestinal side effects are common, they are generally mild and transient, especially with proper patient education and dose titration. Current evidence supports a favorable safety profile in the IBD population, warranting further investigation through ongoing clinical trials.

Desai et al. found that both UC and CD patients with DM2 presented a reduced risk of surgery when treated with GLP-1 RAs compared to other hypoglycemic agents (97). A Danish nationwide cohort study also reported a lower risk of corticosteroid use and hospitalization in IBD patients with DM2 undergoing GLP-1 RAs rather than other antidiabetic treatments (98). Additionally, a nationwide study conducted in Israel found improved IBD outcomes, with a significant reduction of hospitalization rates; however, these benefits were limited only to obese patients (99). Furthermore, recent finding reported that GLP-1 RAs significantly reduced C-reactive protein (CRP) levels in obese IBD patients, alongside a nearly statistically significant reduction in fecal calprotectin (100). However, not all studies align with these results. Levine et al. observed no statistically significant differences in disease exacerbation, corticosteroid-free remission, or therapy escalation in the same cohort of 224 IBD patients after one year of GLP-1 RAs treatment. Despite this, CRP levels showed improvement (101). A further study conducted on IBD patients and not diagnosed with DM2 also reported no significant alterations in inflammatory markers. However, only a small number of patients were included in the study, and median levels were not elevated even before the commencement of GLP-1 RAs’ treatment (102). These discrepancies may stem from short observation periods and small sample sizes respectively (101, 102).

With regard to safety concerns, the most prevalent adverse effects associated with GLP-1 RAs are gastrointestinal, such as bloating, dyspepsia, nausea, vomiting, diarrhea and constipation (107). It is noteworthy that the majority of these gastrointestinal manifestations are of a mild nature and predominantly occur during the titration phase (108). These symptoms are often the reasons why patients stop their treatment. Registration clinical trials show that 16-37% of patients discontinue within a year (109–111). However, real-life analyses indicate a higher discontinuation rate, of approximately 70% stopping within 2 years, especially in non-diabetic patients (112, 113). Interestingly, GLP-1 RAs showed a favorable safety profile in IBD patients, exhibiting comparable tolerability to non-IBD populations. Clarke et al. found that IBD did not affect weight loss outcomes in obese patients and that anti-TNF-α therapy did not reduce the likelihood of achieving ≥5% total weight loss (TWL) (66% vs. 58%, P = 0.33). This indicates that the combination of these agents can be safely and effectively administered to patients with IBD (103). Moreover, IBD patients exhibited lower prevalence of nausea, vomiting, and diarrhea, but increased rates of constipation (11%) in comparison to the general population (103). Two retrospective studies further corroborated the safety and efficacy of GLP-1 RAs in treatment of obesity in IBD patients. They reported mild gastrointestinal symptoms and no substantial changes in disease activity scores (104, 105). Semaglutide has been observed to induce the most substantial weight loss in IBD patients, with no discrepancies in the attainment of >5% TWL when compared to the general population (104–106). Additionally, semaglutide has been shown to have a comparable risk profile to other GLP-1 RAs with respect to gastrointestinal adverse effects in the IBD population (106). Consequently, some authors consider GLP-1 RAs to be safe in the IBD population, however, they emphasise the necessity of providing educational advice to patients (e.g. small and frequent meals) and employing a gradual dose-up titration strategy (114).

Currently, two ongoing clinical trials are investigating the role of GLP-1 RAs in IBD management, that would lead to further knowledge: a French study (ID NCT05196958) evaluating the safety and efficacy of GLP-1 RAs in treatment of DM2 in overweight IBD patients (115), and an American study (ID NCT06774079) comparing the efficacy of GLP-1 RAs tirzepatide versus diet in CD patients (116).

In summary, the interplay between IBD and metabolism represents a growing area of research and therapeutic interest, with GLP-RAs emerging as a promising therapeutic option. While preclinical and clinical studies have reported anti-metabolic and anti-inflammatory benefits with a favourable safety profile, further evidence from long-term and large-scale trials is necessary to guide clinicians in real-life scenarios.

## 5 Conclusions

In conclusion, GLP-1 plays a pivotal role in controlling both blood glucose levels and body weight. The interaction between GLP-1, IELs and gut microbiota highlights its vital role in preserving the integrity of the intestinal mucosal barrier and the gut immunity homeostasis. Emerging evidence suggests the potential benefits of GLP-1 RAs in the treatment of IBD through enhanced mucosal healing and reduced inflammation. Furthermore, GLP-1 RAs seem to have similar safety profile in the IBD population to the one observed in the general population, based on real-world observations. However, further research is required to ascertain the long-term outcomes of GLP-1 RAs in IBD patients, with some studies indicating potential benefits and others highlighting concerns regarding altered gut immunity. Further clinical evidence is needed to clarify their role, optimise treatment strategies, and assess their impact on disease progression and patient outcomes.

However, further research is required to ascertain the long-term outcomes of GLP-1 RAs in IBD patients, with some studies indicating potential benefits and others highlighting concerns regarding altered gut immunity. Further clinical research is needed to clarify their role, optimise treatment strategies, and assess their impact on disease progression and patient outcomes.
